# Bat Rabies, Texas, 1996–2000

**DOI:** 10.3201/eid1005.030719

**Published:** 2004-05

**Authors:** Rodney E. Rohde, Bonny C. Mayes, Jean S. Smith, Susan U. Neill

**Affiliations:** * Texas State University at San Marcos, San Marcos, Texas, USA; † Austin Community College, Austin, Texas, USA; ‡ Centers for Disease Control and Prevention, Atlanta, Georgia, USA; § Texas Department of Health Bureau of Laboratories, Austin, Texas, USA

**Keywords:** Rabies prevalence, bats, molecular epidemiology, wildlife reservoir, monoclonal antibody, surveillance, bat rabies, RT-PCR

## Abstract

Bats submitted to the Texas Department of Health (1996–2000) were speciated and tested for rabies virus antigen by direct immunofluorescence microscopy. Antigenic analysis of rabies virus–positive specimens was performed with monoclonal antibodies against the nucleoprotein of the virus; atypical or unexpected results were confirmed by genetic analysis of nucleoprotein sequence.

Most information on bats as reservoirs for rabies virus (RABV) is obtained from animals submitted by the public to local health departments for rabies testing. These data are limited by the following factors: 1) most bat submissions are from a few species found around human dwellings and outbuildings; little is known about rabies in the >30 bat species whose habitats are restricted to forest, desert, and mountainous areas (*1*,*2*); and 2) few state laboratories identify their bat submissions to species, and fewer still have the resources to collect data on the incidence and prevalence of different antigenic and genetic variants of RABV (RABVV) (*3*).

The Texas Department of Health laboratory receives 600–1,300 bats each year for rabies testing. Approximately 11% of the bats submitted test positive for RABV. All are identified to species, and all RABV-positive specimens are typed with a panel of monoclonal antibodies (MAbs) to determine the antigenic variant of rabies. Samples from bat species uncommonly found rabid in Texas or from more common species infected with atypical virus variants are submitted to the Centers for Disease Control and Prevention (CDC) for nucleotide sequence analysis.

The objectives of this study were to determine the status of state surveillance for bat-associated rabies at the species level, assess the comparative characteristics of the antigenic and genetic variants of rabies in bats in Texas, and examine the need for bat speciation and genetic variant determination in assigning uniform variants of RABV.

## The Study

All bats submitted to the Texas Department of Health Rabies Laboratory for RABV testing from 1996 to 2000 (n = 3,989) were used in this study. All bats were either identified upon receipt or frozen and saved for future speciation. A key based on external characteristics of adult bats from The Bats of Texas (*4*) was used to make initial determinations. Species identifications were confirmed by comparing specimen data with the more detailed descriptions in that book. All bats with uncertain identifications were taken to Bat Conservation International for clarification. Bats were shipped to Texas Tech University for species confirmation.

Brain tissues from RABV-positive bats were tested by direct immunofluorescence (Centocor, Malvern, PA; Chemicon, Temecula, CA) for their reaction with MAbs against the nucleoprotein of the RABV (*5*). MAbs were provided by CDC and have been used extensively to identify RABVV (*1*,*6*–*10*).

RNA in brain material was extracted with TRIzol, according to the manufacturer’s instructions, then reverse transcribed and amplified by polymerase chain reaction using primers 10g and 304 (*11*). Amplicons purified by using the Wizard TM Minipreps DNA purification system (Promega, Madison, WI) were sequenced with the ABI PRISM DNA Sequencing Kit (PE Applied Biosystems, Foster City, CA), according to manufacturer’s instructions. Automated fluorescence sequencing was performed on an Applied Biosystems 310 DNA sequencer (PE Applied Biosystem). Nucleotide sequence from a 302-bp region of the RABV nucleoprotein (bp 1175 to 1476) was aligned with Pasteur RABV, GenBank accession no. M13215 (*12*). Nucleotide sequence for Texas bat samples was compared to the 17 genetic lineages of RABV identified for bat samples in a CDC repository (GenBank accession nos. AF045166, AF394868-394888, and AY039224–39229) (*13*). A phylogenetic analysis of the sequence data was conducted by using the programs DNADIST, NEIGHBOR, SEQBOOT, and CONSENSE in the PHYLIP package, version 3.5 (*14*). Graphic representation of the phylogenetic analysis was obtained with the program TREEVIEW (*15*).

During the 5-year study period, 3,989 bats were submitted for RABV testing. More than 96% (n = 3,830) of all bats submitted from 1996 to 2000 were easily speciated; 159 (3.8%) were too decomposed, damaged, or immature for reliable identification to species or were inadvertently discarded before identification was complete. This dataset includes representatives from 19 of the 32 species found in Texas; also included are *Desmodus rotundus* from the Fort Worth Zoo and one or two species of fruit bats. *Tadarida brasiliensis* was the most common species submitted for testing, followed by *Lasiurus borealis*. Rare submissions include *Mormoops megalophylla*, *Myotis austroriparius*, *M. californicus*, *M. ciliolabrum*, *M. thysanodes*, *M. yumanensis*, *Antrozous pallidus*, and *Nyctinomops macrotis*. The prevalence of RABV in the submitted samples remained fairly constant; prevalence ranged from 8.9% in 1998 to 12.4% in 1997 with an average prevalence of 11%. Specimens from nine of the species tested positive for RABV. *L. cinereus* had the highest average positivity rate (26.3%) followed by *T. brasiliensis* (16.4%); this finding is in agreement with results of a recent study of the continental United States (*16*). *N. humeralis* had the lowest average positivity rate (0.7%) (Table 1).

**Table 1:** Bat species submitted to the Texas Department of Health laboratory for rabies virus (RABV) testing (1996–2000)

Species	Total no. received (1996–2000)	No. testing positive for RABV (%)
*Antrozous pallidus* (pallid bat)	3	0
*Desmodus rotundus* (vampire bat)^a^	4	0
*Eptesicus fuscus* (big brown bat)	14	1 (7.1%)
*Lasiurus borealis* (eastern red bat)	714	48 (6.7%)
*L. cinereus* (hoary bat)	57	15 (26.3%)
*L. ega* (southern yellow bat)	80	2 (2.5%)
*L. intermedius* (northern yellow bat)	153	14 (9.2%)
*Lasionycteris noctivagans* (silver-haired bat)	5	0
*Lasiurus seminolus* (seminole bat)	14	2 (14.3%)
*Mormoops megalophylla* (ghost-faced bat)	1	0
*Myotis austroriparius* (southeastern myotis)	1	0
*M. californicus* (California myotis)	1	0
*M. ciliolabrum* (western small-footed myotis)	1	0
*M. thysanodes* (fringed myotis)	1	0
*M. velifer* (cave myotis)	172	4 (2.3%)
*M. yumanensis* (yuma myotis)	1	0
*Nycticeius humeralis* (evening bat)	410	3 (0.7%)
*Nyctinomops macrotis* (big free-tailed bat)	5	0
*Pipistrellus subflavus* (eastern pipistrelle)	40	0
*Tadarida brasiliensis* (Brazilian free-tailed bat)	2,062	338 (16.4%)
Fruit bats, not speciated^b^	2	0
Juvenile yellow bats (*L. ega* or *L. intermedius*)	65	0
*Lasiurus* sp.^c^	24	1 (4.2%)
Unable to identify species^d^	159	6 (3.8%)
Total	3,989	434 (11%)

^^a^^Vampire bats part of captive colony in zoo. ^^b^^Appear to be two different species. ^^c^^Too damaged to determine species; will probably fall within one of the above mentioned *Lasiurus* sp. ^^d^^Extremely damaged, decomposed, or immature.

MAb reaction patterns have been identified, and the complete N gene sequence is available from GenBank for the RABV associated with *T. brasiliensis*, *L. borealis*, *L. cinereus*, *L. intermedius*, and *Eptesicus fuscus* (Table 2 and Figure). Adequate material was available for examination of 407 of 416 rabies-positive samples (1996–2000) from these five bat species in Texas by antigenic analysis; 402 of 407 samples had reaction patterns that were expected for the species. Genetic analysis was used to confirm the antigenic typing result of 14 of the 407 samples typed by antigenic methods; other Texas samples included in the analysis are two samples not submitted to the Texas Department of Health, seven samples from 1986 to 1995, and 10 samples from bat species not commonly found rabid in Texas.

**Table 2:** Monoclonal antibody (MAb) reaction patterns of bat rabies virus variants (RABVV), Texas^a^

Pattern	MAb 1	MAb 12	MAb 19	MAb 7	MAb 13	Bat species associated with RABVV
1	P	P	N	P	P	*Tadarida brasiliensis* (Brazilian free-tailed bat)
2	N	N	N	P	P	*Lasiurus borealis* (eastern red bat)
3	N	W	N	W	P	*L. cinereus* (hoary bat)
4	N	N	N	P	N	*L. intermedius* (northern yellow bat)
5	N	P	P	P	P	*Eptesicus fuscus pallidus* (big brown bat)

^a^P, positive; N, negative; W, weakly positive.

**Figure:**
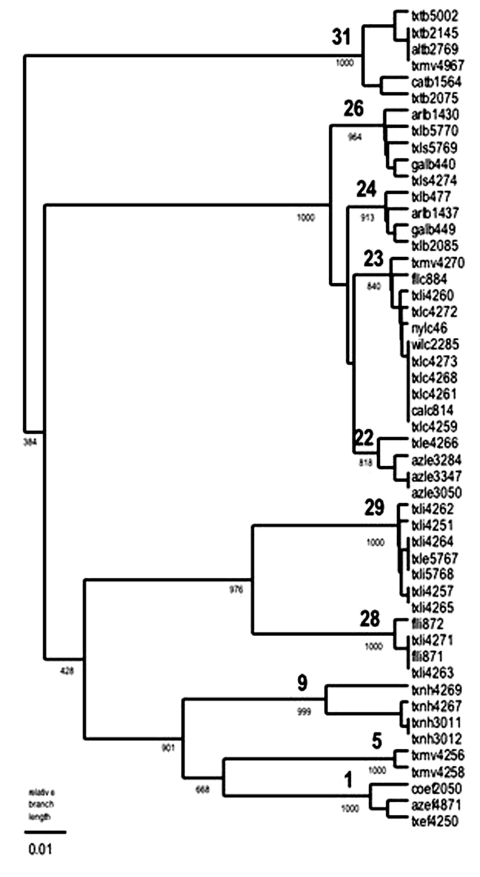
Genetic lineages of rabies virus in Texas bat populations. Lineages were determined by using the unweighted pair group method using arithmetic averages (UPGMA) method on the basis of 302 nucleotides of nucleoprotein gene sequence. Bootstrap values of 1,000 replicates indicate the robustness of the corresponding node. Representatives of each lineage were deposited in GenBank under the following accession numbers: 31, AF394876; 26, AY039224; 24, AF394886; 23, AF394883; 22, AY170247; 29, AY208163; 28, AF394878; 9, AY208164; 5, AY208165; 1, AF394887.

MAb reaction pattern 1 described the RABV in 331 of 332 samples from *T. brasiliensis*. MAb reaction pattern 2 was found in 1 of 332 samples, suggesting interspecific infection through contact with *L. borealis*; however, no RABV genetic material could be amplified from the *T. brasiliensis* sample displaying reaction pattern 2. Reference samples from *T. brasiliensis* collected in Texas in 1984, 1993, and 1998 clustered in lineage 31 with virus from *T. brasiliensis* collected across the range of these bats in the United States.

Reaction pattern 2 described the RABV in 45 of 46 samples from *L. borealis*. MAb reaction pattern 1 was found in 1 of 46 samples, suggesting interspecific infection through contact with *T. brasiliensis*; however, the finding was not confirmed by genetic analysis. This sample, txlb 5770, clustered in lineage 26 with reference samples of *L. borealis* collected in Texas in 1986 and virus from *L. borealis* collected elsewhere in the eastern United States.

Reaction pattern 3 described the RABV in 13 of 14 samples from *L. cinereus*. *L. cinereus* samples displaying reaction pattern 3 (n = 4) clustered in lineage 23 with *L. cinereus* samples collected across the range of these bats in the United States. One *L. cinereus* sample (txlc4259) differed in its reaction with the MAb panel (1-n, 12-w, 19-n, 7-w, 13­w), but genetic analysis showed the sample clustered with other *L. cinereus* samples in lineage 23.

Reaction pattern 4 was identified in 12 of 14 RABV from *L. intermedius*. The reaction pattern of the two exceptional *L. intermedius* samples was not known to be associated with any bat species. Because the CDC repository contains only Florida *L. intermedius* samples, seven Texas *L. intermedius* samples of reaction pattern 4 were submitted for genetic analysis. Five of the seven samples clustered in lineage 29, a lineage new to the CDC repository and not in GenBank. Two of the seven samples clustered in lineage 28 with *L. intermedius* samples from Florida. The two Texas *L. intermedius* samples with unrecognizable MAb patterns (1-w, 12-n, 19-n, 7-w, 13-n; 1­n, 12-w, 19-n, 7-w, 13-p, respectively) clustered in lineage 29 (txli5768) and lineage 23 (txli4260) by genetic analysis.

The single RABV sample from *E. fuscus* displayed reaction pattern 5. This sample was unavailable for genetic analysis, but a 1994 Texas *E. fuscus* sample (txef4250) with reaction pattern 5 RABV shared 99% identity with RABV samples from western big brown bats in lineage 1 (shown as representative samples from Colorado and Arizona).

The remaining 11 rabies-positive samples from Texas bats were collected from *M. velifer*, *L. seminolus*, *L. ega*, or *N. humeralis.* Because no MAb reaction patterns or genetic lineages have been established for these species, 10 of 11 samples were typed by antigenic and genetic methods. The MAb reaction pattern for one sample from *N. humeralis* was determined, but the sample was unavailable for genetic analysis.

The four samples from *M. velifer* displayed three different MAb reaction patterns. Sample txmv4267 displayed reaction pattern 1 and also clustered with other samples from *T. brasiliensis* in lineage 31 in the genetic analysis. The MAb reaction pattern of sample txmv4270 is not known to be associated with any bat species (1-n, 12-n, 19-n, 7­n, 13-p), but the genetic analysis showed an association with *L. cinereus* in lineage 23. MAb reaction pattern 2 was found in txmv4258; however, genetic typing indicated lineage 5, a lineage new to the CDC repository. Lineage 5 was also found in sample txmv4256, which had displayed an MAb reaction pattern not known to be associated with any bat species (1-n, 12-p, 19-n, 7-w, 13-p). No other samples of lineage 5 exist in the CDC repository, and the repository contains only one other sample from *M. velifer* (from California). The California *M. velifer* sample clustered with *T. brasiliensis* samples in lineage 1 (not shown).

The *N. humeralis* samples (n = 3) had previously unrecognized yet identical reaction patterns (1-n, 12-p, 19-n, 7-p, 13-n), as did two reference samples from this species collected in 1995. Two of the 1998 *N. humeralis* samples (txnh4267 and txnh4269) and two reference samples (txnh3011 and txnh3012) indicated lineage 9, a lineage new to the CDC repository; that repository contains only one other sample of a lineage 9 RABV, an *M. austroriparius* from Florida (not shown). The only additional RABV sample from *N. humeralis* in the repository, also from Florida, clustered with *L. borealis* samples in lineage 26 (not shown).

The two RABV samples from *L. seminolus* displayed MAb pattern 2, associated with *L. borealis*. Both samples (txls4274 and txls5769) clustered with RABV from *L. borealis* in lineage 26. Three additional samples from *L. seminolus* in the CDC repository (all from Florida) also clustered with *L. borealis* samples (not shown).

The *L. ega* sample (txle4266) displayed a unique MAb pattern (1-p, 12-n, 19-n, 7­p, 13-p) and clustered in lineage 22 with three samples from *L. ega* bats from Arizona. Four additional samples from *L. ega* in the CDC repository did not contain a lineage 22 RABV. These samples contained lineages 1 and 23, which suggests infection through contact with *T. brasiliensis* and *L. cinereus*, respectively.

## Conclusions

For those laboratories without genetic typing capability, antigenic analysis with MAbs offers a rapid, simple, and inexpensive means of typing RABV for epidemiologic surveys. Our study suggests MAb typing can be useful for large-scale surveys in which hundreds to thousands of virus samples originate from only one or two bat species and the question is simply “Do we find in these species the RABVV that we expect to find?” All but 5 of 407 samples from *T. brasiliensis,* *L. borealis*, *L. cinereus*, *L. intermedius*, and *E. fuscus* tested in this study displayed the MAb patterns expected for the species. However, MAb typing by fluorescence microscopy lacks precision. Surveys that rely solely on antigenic typing underestimate the true diversity of RABV in bat populations and may oversimplify rabies transmission cycles. For example, antigenically identical samples from both *L. borealis* and *L. intermedius* segregate as two different genetic lineages (Figure) (*13*). This pattern of divergence does not correlate with time or the area in which either species was collected and must reflect some as-yet unknown aspect of natural history that partitions and segregates virus populations. These findings suggest not only that genetic typing offers a more precise identification of a RABVV but also that genetic analysis of RABV may help us better understand how the natural history of the host drives viral evolution.

The observed genetic diversity among the 23 samples sequenced for this study was unexpectedly large for such a small sample set. Two lineages (5 and 29) consisted solely of Texas samples; lineage 9 had been identified previously in only one other sample (an *M. austroriparius* from Florida); and lineage 22 had been identified previously only in *L. ega* samples from Arizona. The small number of samples in these four lineages does not allow designation of reservoir status for these species, but the genetic diversity in the RABV in Texas reflects the diversity of bat species in the southwestern United States and suggests that many, if not all, bat species transmit distinctive RABV. Identification of the species association of different variants of RABV could lead to valuable information about routes of virus transmission and mechanisms by which RABV persists in different bat populations.
